# Women Are Seen More than Heard in Online Newspapers

**DOI:** 10.1371/journal.pone.0148434

**Published:** 2016-02-03

**Authors:** Sen Jia, Thomas Lansdall-Welfare, Saatviga Sudhahar, Cynthia Carter, Nello Cristianini

**Affiliations:** 1 Department of Computer Science, University of Bristol, Bristol, United Kingdom; 2 School of Journalism, Media and Cultural Studies, Cardiff University, Cardiff, United Kingdom; University of Hertfordshire, UNITED KINGDOM

## Abstract

Feminist news media researchers have long contended that masculine news values shape journalists’ quotidian decisions about what is newsworthy. As a result, it is argued, topics and issues traditionally regarded as primarily of interest and relevance to women are routinely marginalised in the news, while men’s views and voices are given privileged space. When women do show up in the news, it is often as “eye candy,” thus reinforcing women’s value as sources of visual pleasure rather than residing in the content of their views. To date, evidence to support such claims has tended to be based on small-scale, manual analyses of news content. In this article, we report on findings from our large-scale, data-driven study of gender representation in online English language news media. We analysed both words and images so as to give a broader picture of how gender is represented in online news. The corpus of news content examined consists of 2,353,652 articles collected over a period of six months from more than 950 different news outlets. From this initial dataset, we extracted 2,171,239 references to named persons and 1,376,824 images resolving the gender of names and faces using automated computational methods. We found that males were represented more often than females in both images and text, but in proportions that changed across topics, news outlets and mode. Moreover, the proportion of females was consistently higher in images than in text, for virtually all topics and news outlets; women were more likely to be represented visually than they were mentioned as a news actor or source. Our large-scale, data-driven analysis offers important empirical evidence of macroscopic patterns in news content concerning the way men and women are represented.

## Introduction

Researchers have consistently argued and demonstrated the ways in which news media representations help to shape public perceptions about the world [1,2] including those around gender [3]. For example, various studies have investigated how women are under-represented in the news media [4,5,6,7], looking at the percentage of the news written by women [5], how often women are mentioned in news stories and how often women are used as experts [6,7], with other studies covering the images displayed in the news [4]. These studies, amongst many others, have established how males dominate the narrative of mainstream news media. However, most of this research has been performed by hand on small samples of news articles, examining a limited number of news outlets over short time periods. Critical investigation of news output can now be undertaken, including both text and images, on a vast scale using modern Artificial Intelligence (AI) technologies. Our study is one of only a few using systematic large scale data collection focusing on issues around gender representation in the news [8,9,10,11]. The use of a large number of articles, obtained from hundreds of different outlets, allows this study to be less dependent on the specific choice of outlets, and also gives us sufficient statistical power to analyse the link between gender ratio (the ratio between males and females represented in an article) and certain topics, including entertainment, fashion, religion, business and politics.

Utilising these technologies, in this paper we report on our analysis of over two million news articles collected over a six month period from hundreds of English language online news outlets, extracting the mentions of male and female named persons and classifying male and female faces in the images associated with them. The question we want to address is whether there is some pattern in the way gender representations relate to topic or mode of communication (i.e., image versus text), and if these can reveal something about the cultural mechanisms behind differences in gender representation.

We present results broken down by topic category, news outlet and mode of content, confirming the predominance of males in almost every topic and news outlet. Importantly, the study also reveals how there was a significant structure to the ways in which gender inequalities were represented, with females more likely to appear in photographs and other illustrative material than in written news text.

This finding was consistent across nearly all topics and in over 96% of the news outlets under scrutiny, and points in the direction of a traditional association in western philosophy, linking women to bodies and the private sphere [12], and men to mind and the public sphere. More importantly, it lends support to the longstanding claim by feminist scholars that women’s voices are marginalised in the media, a fact that has significant implications for democracy [6,13].

## Materials and Methods

### 3.1 Data Collection

We collected 2,353,652 news articles from over 950 news outlets that have a web presence, spanning a period of six months from when collection of news images began, covering 19^th^ October 2014 to 19^th^ April 2015. News articles were gathered from the Really Simple Syndication (RSS) feed for the front page of the news outlets, representing the top stories on their website, an analogous concept to the front page of a newspaper. Those written in a language other than English were additionally filtered out of the corpus, bypassing issues associated with working in a multilingual setting. Collection of the articles was performed by our modular system for news media analysis [14], using a combination of web crawler modules and a web scraper module, which have previously been used for various large-scale analyses of news media content, including detecting the framing of scientific coverage in the media [15], detecting structure in the choice of stories covered by news outlets across Europe [16], and large-scale analysis of topic, style and gender imbalance in news text content [11].

#### 3.1.1 Web crawling

The first web crawler module in the system was used to identify RSS feeds on the web that potentially belong to news media outlets starting from a number of seed web pages, such as a list of newspapers within the United Kingdom on Wikipedia [17] or from the homepage of news outlets. The list of RSS feeds found using this process was then inspected by hand to find RSS feeds belonging to news outlets. Each RSS feed belonging to a news outlet was manually annotated with the content language of the feed, the country location of the outlet, and a label designating if it is the main RSS feed for the news outlet, representing the news published to the front page of the news outlet’s website.

A second web crawler module continuously crawled the list of RSS feeds that had been labelled as the main feed for the news outlet in order to discover any news articles that had not been previously seen by the system. Articles were compared against all previously collected articles using a hashed version of the title and Uniform Resource Locator (URL) of the news article to prevent duplicate articles being collected.

#### 3.1.2 Web scraping

Once a news article was identified, we retrieved the full text content of the news article and its associated main image from the web page specified by the URL in the RSS feed. The main text content of the article was then identified and separated from other non-content textual information (such as comments, related stories or web page navigation) within the web page by extracting the largest set of text with a common parent within the web page HTML structure, an adaption of the text-to-tag ratio presented in [18]. This ratio was previously validated against a manually extracted gold standard, achieving a mean edit distance ratio of 94.19%, where the edit distance ratio is the percentage of keystrokes that would be required to transform the automatically extracted text into the manually extracted text.

The main image associated with each article was extracted by identifying the image specified in the meta-information from the web page header, corresponding to the main image the news outlet had chosen for the article. Using this method allowed us to avoid problems when many images are present on the web page which are not necessarily related to the news article itself (such as from related stories) by only collecting images which the news outlet have specifically labelled as best representing the news article. We also recorded each time an article did not specify an image in the web page header (i.e. no URL), or if the URL did not resolve to an image, for example if the image no longer exists or has been moved from its original location (i.e. missing image).

### 3.2 Content Analysis

For each news article in the corpus, we used automated methods to extract information from the text and image, annotating each news article with topic categories, named person entities from the text and the gender of faces in the images.

#### 3.2.1 Topic classification

We used Support Vector Machines (SVMs) [19] trained for high precision on the well-known Reuters [20] and New York Times [21] news corpora, along with online linear perceptron models [22] trained on news media within our modular architecture [14] to classify news articles into 12 topic categories as defined by the editors of Reuters and the New York Times. The SVM and perceptron algorithms both work by learning a model, represented in this case as a set of weights assigned to different words, that can best separate the content of articles labelled as belonging to a topic (positive class) from those which do not (negative class). The learnt model can then be applied to each news article in turn, assigning a topic score to each article based upon the weighted sum of the words it contained.

The performance of the topic classifiers was assessed on the Reuters and New York Times corpora, with precision ranging from 73.5% (Environment) to 98.3% (Sports). In total, our automated methods for topic classification assigned 62.7% of the news articles to one of 12 topic categories, with each annotated news article belonging to an average of 1.07 topic categories. News articles not assigned to any of the 12 topic category were labelled as “Other”.

#### 3.2.2 Entity extraction

We extracted person named entities from the full text of the articles using the ANNIE plugin of GATE [23] followed by a series of steps aimed at improving the quality of co-reference resolution and gender classification based upon the methodology of [24]. Each named entity had a set of co-references (alternative representations of the entity) which we used to generate a co-reference network, where the nodes of the network represent entities and the edges correspond to the number of articles the entities were seen together as co-references. The co-reference network was then pruned using minimum thresholds for the name length, number of different articles and news outlets the entity occurred in, along with a maximum threshold for the number of alternative representations. Entities were extracted as the remaining connected components, using the most frequently occurring representation as the entity name. The gender for each entity was obtained by taking the majority label assigned by GATE in the named entity recognition step.

We validated our entity extraction and gender classification against manually annotated labels for 1,000 extracted entities, finding that 95.6% of the entities refer to a person name, and that for those person names, we correctly identify the gender for 97.1%, using the manually annotated labels as a gold standard.

#### 3.2.3 Face detection and gender classification

We first identified images that contain a face using the Viola-Jones algorithm [25] implemented in OpenCV [26]. This algorithm extracted Haar-like features from the image, calculated from the difference between neighbouring patches of pixels within the image, before it selects the features which are most useful for face detection using AdaBoost.

Once an image had been identified as containing a face, we extracted features known as local binary patterns (LBP), which have been shown to provide efficient descriptors for facial images [27] and are computed for each pixel in an image by assigning a binary number to the pixel based upon the value of pixels in its neighbourhood falling above or below the centre pixel’s value. These LBP features were used to train an online variant of SVMs, known as C-Pegasos, in order to classify the gender of the face images [28]. C-Pegasos works in a way similar to the SVM and perceptron algorithms used for topic classification, but in this case learns weights for each of the LBP features, rather than for a set of words as was performed for topic classification.

The performance of this process for gender recognition had previously been assessed on the Labeled Faces in the Wild (LFW) dataset [29] and was shown to be balanced across genders, obtaining an accuracy of 96.37%.

#### 3.2.4 Annotated article statistics

Using these high precision methods, we were able to extract 1,376,824 images from 973 news outlets, with 802 of the news outlets containing a total of 2,171,239 references in 1,065,770 news articles to 116,862 unique people in the text. Table 1 details the number of news articles that were annotated with extracted images, entities and topics using these methods.

**Table 1:** Number of news articles that were annotated with extracted images, entities and topics. At each step of our extraction process, we use high precision computational methods in order to ensure that the information we extract from the news articles is of high quality. As such, we do not extract faces, entities or topic from every single news article.

Label	Description	Count
No Image	No image URL present in the news article header meta information.	846,980
Missing Image	No article image could be retrieved from the URL.	129,848
No Face	The article image does not contain a face.	904,638
Face	The article image does contain a face.	472,186
No People	No person entities could be fully resolved within the full text of the article.	1,287,882
People	Person entities could be fully resolved within the full text of the article.	1,065,770
No Topic	No topic category was assigned to the news article.	878,683
Topic	One or more topic categories were assigned to the news article.	1,474,969

## Results

In this study, we sought to investigate how the gender representation differs across topic categories, modes of content (text or images) and by news outlets. Our first set of measurements split the data into each of the 12 topic categories, or the “Other” category, and measured the gender balance in the text and images for each topic. In the second set of measurements, we split the data based upon the news outlet that published the article, presenting results focused on popular outlets with high readership, and a sufficiently large number of news articles present in our corpus to be representative.

### 4.1 Topic Gender Balance

Examining gender balance in the corpus of news articles analysed, we focused on how the representation of men and women featured in the news changed when examining the topic category of the news article, along with any differences between the main text of the article and the image associated with it. For each of the 12 topic categories, plus the additional “Other” category, we computed the probability that an entity or face image was classified as male.

**Fig 1:**
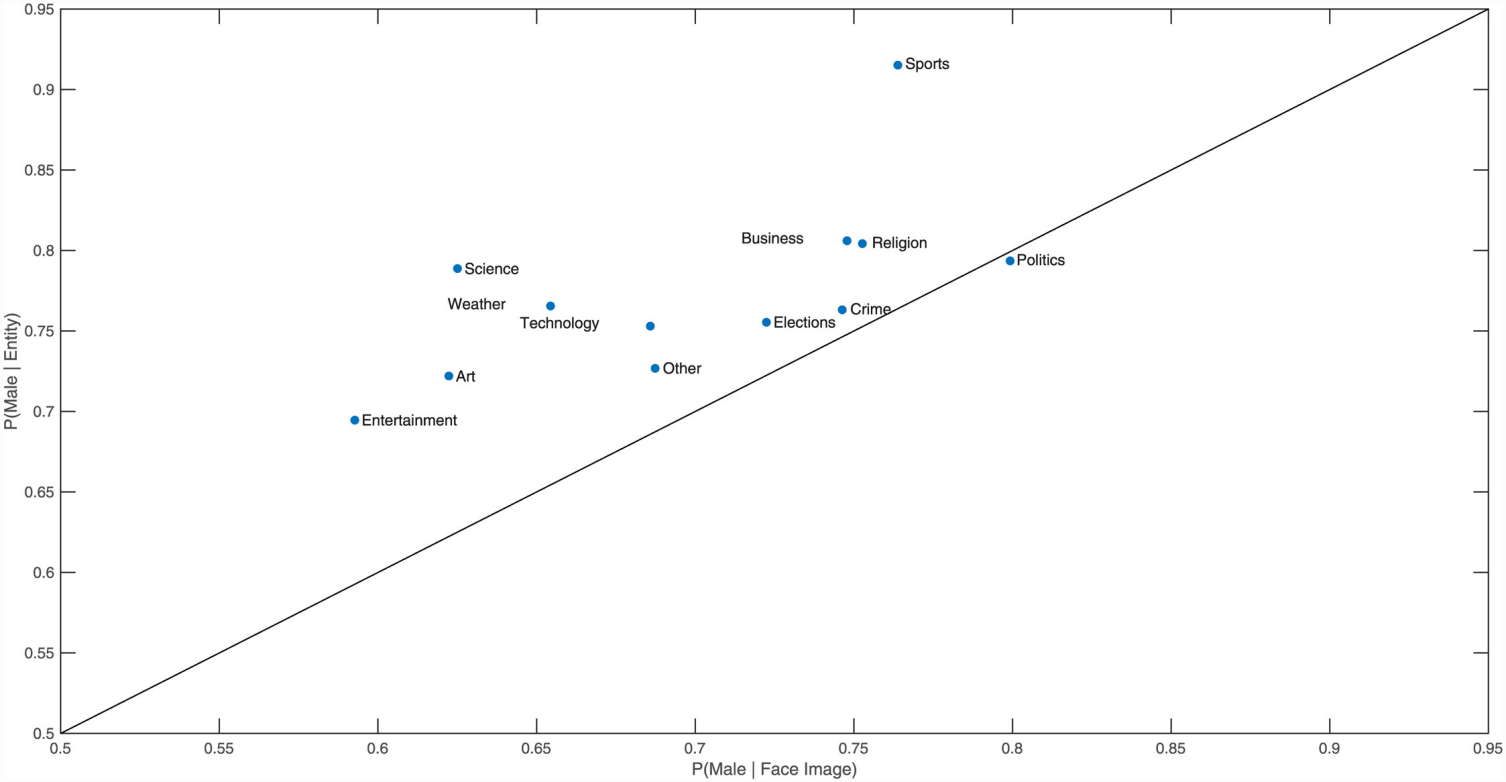
Gender balance for each topic category. Fashion was excluded for readability, and would reside at (36.1,45.9). All topics above the diagonal have a higher probability of a face being female in an image than a person entity in the text being female.

We found that across all topic categories except Fashion, mentions of males dominated in written texts, with the probability of an entity being male ranging from 69.5% in Entertainment to 91.5% in Sports. The results were similar for images, where the probability of a face image being male ranged from 59.3% in Entertainment to 79.9% in Politics. Fig 1 shows the gender composition for each topic category excluding Fashion, with points above the diagonal indicating when a topic exhibits less by way of gender balance (in favour of males) in the mention of entities in the text, while a point below the diagonal indicates a topic where there is less balance in the face images of the topic. We can see that there was only one topic, Politics, in which females were more likely to be mentioned in the text than seen in the image of the news article.

Fashion was found to be the only topic category where mentions of females in the text, or images of female faces, were more likely than those of their male counterparts, with the probability of an entity being male in Fashion equalling 45.9%, while the probability of a face image being male was 36.1%.

Our findings here are supported by manual studies [4,5,6,7], and earlier automated studies [8,9,10], including our own work [11] where we previously found that the ratio of males to females of the most mentioned entities found that Sports was contained the least balanced among the 1,000 most mentioned entities, with the topic of Fashion being the closest to parity.

### 4.2 Outlet Gender Balance

We also wanted to investigate how the balance of males and females featured in the news might change when examining the news outlet that published the news article. For each, we computed the probability that an entity or face image was classified as male, given that the news article they are extracted from was published by the news outlet's main feed.

We focused our results on those news outlets with highest readership and a minimum of 500 face images and entities within the corpus. Readership for each outlet was determined using their global Alexa rank, a measure of the website’s popularity, calculated using a combination of average daily visitors to the website and page-views on the website over the past 3 months [30].

**Fig 2:**
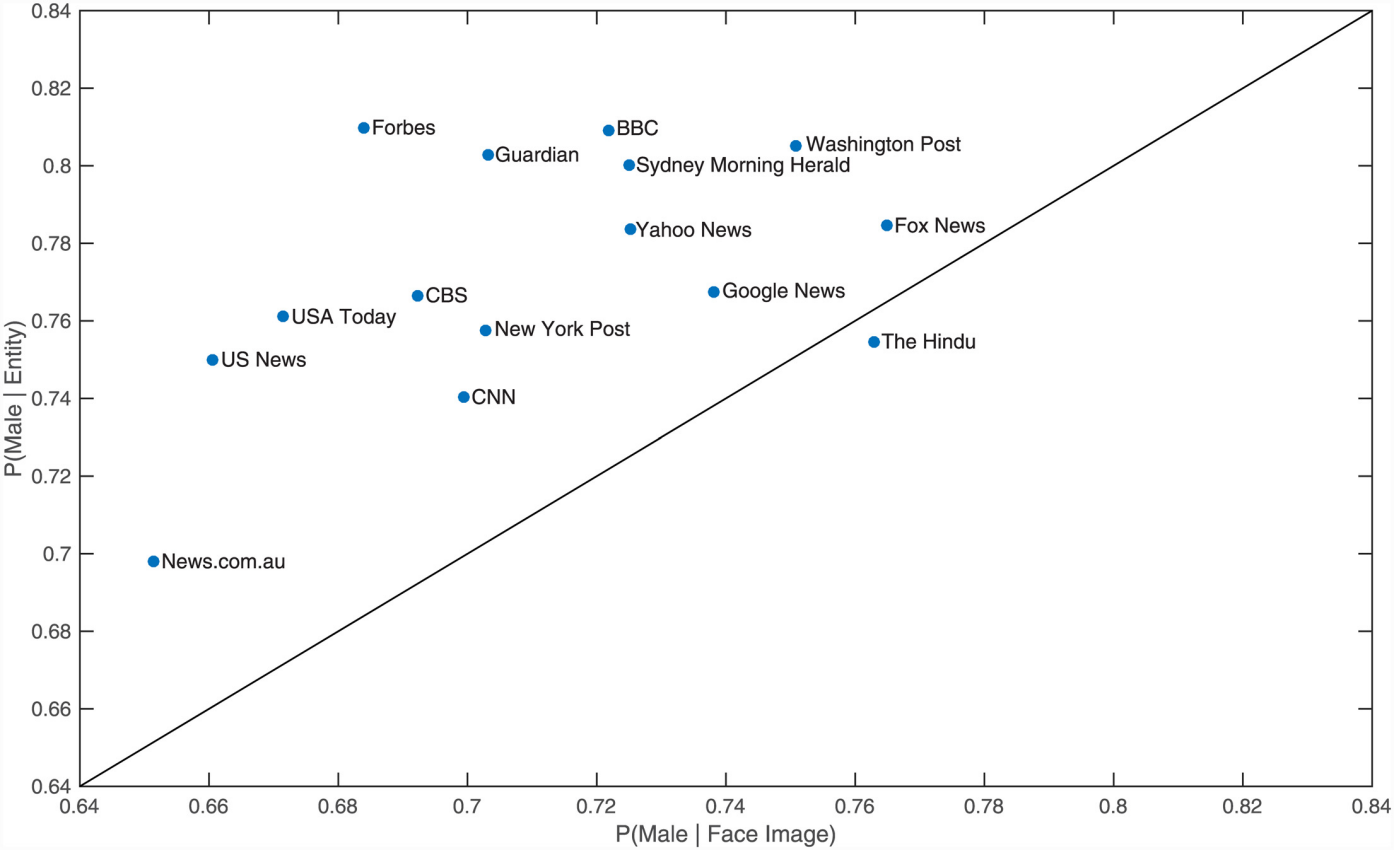
Gender balance for each of the 15 news outlets. All outlets above the diagonal have a higher probability of a face being female in an image than a person entity in the text being female. For one particular outlet displayed (“The Hindu”), the position might not reflect its actual probability of a given entity being male in textual data (vertical axis) due to our Named Entities recognizer having worse performance than average on that outlet. We have included it in the Figure for completeness.

From the top 15 news outlets for which we have data, we found that Forbes was the least balanced of the news outlets in the mention of entities in the text, with 81.0% being males, closely followed by the BBC at 80.9%. Fox News was the least balanced in the choice of images containing females published, with the probability of a face image being male equalling 76.5%. Of the 15 outlets, News.com.au was the closest to gender balance, with the probability of an entity and a face image being classified as male being 69.8% and 65.1% respectively. Fig 2 displays the gender balance for each of the top 15 news outlets, showing that for all of these outlets, they are more likely to show an image of a female than they are to mention one in the text of the news article.

**Fig 3:**
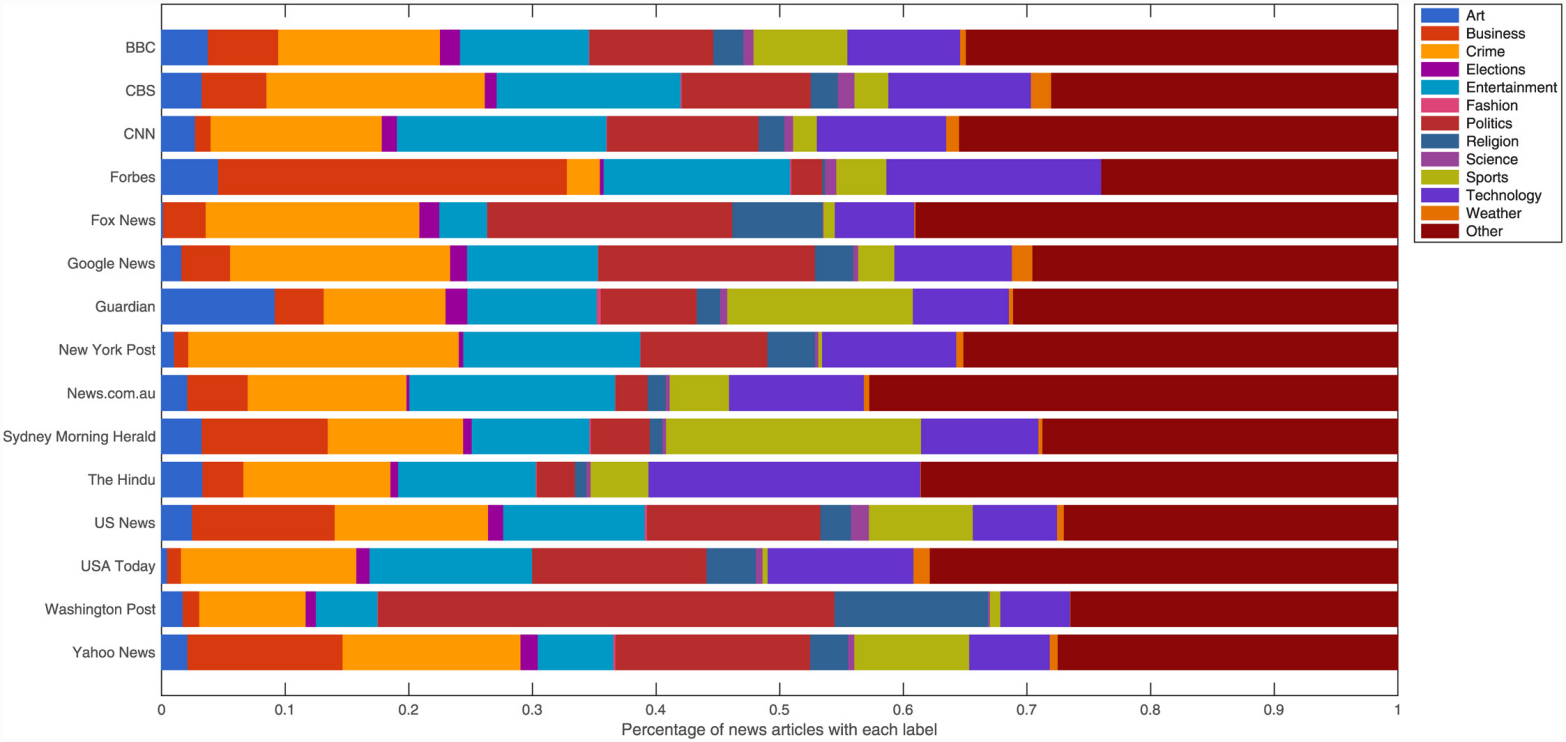
Topic distribution for each of the news outlets. Articles from each news outlet were classified into one of 12 topic categories or the “Other” category. The percentage of articles in each topic are shown for the 15 news outlets in this study.

**Fig 4:**
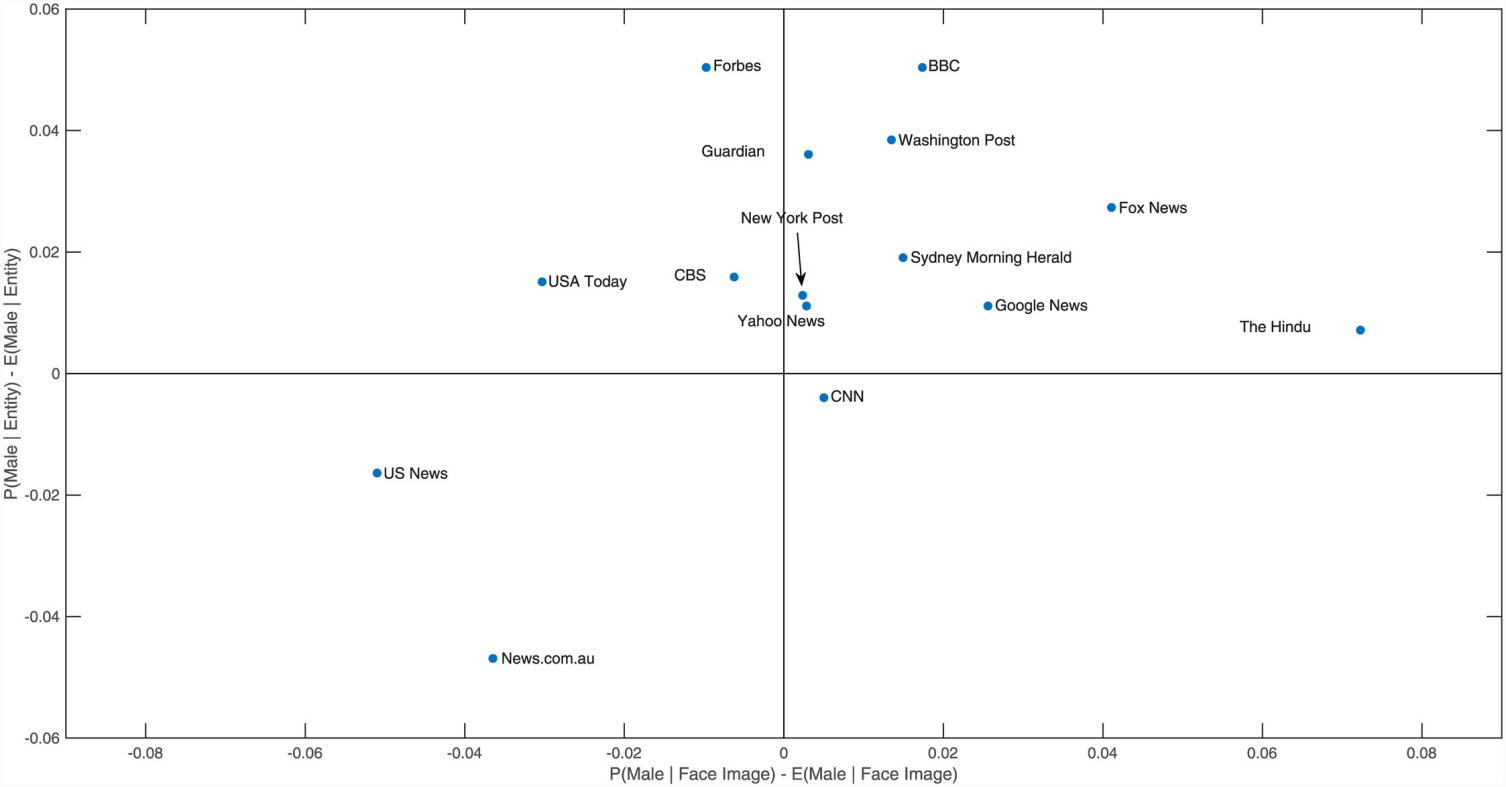
Difference between the actual gender balance and the expected gender balance given the topic distribution of each outlet. Outlets near the origin can explain their gender balance as an effect of their choice of topic coverage. Outlets far from the origin have a gender balance that is not explained by their choice of topic coverage.

We additionally wished to investigate if the differences in choice of topics covered by the news outlets could account for the differences in gender balance. For example, does Forbes have much less gender balance than News.com.au because it is more business and politics (relatively male-dominated topics) focussed, while the latter covers a greater selection of lighter topics, such as entertainment, which are relatively less male-dominated? To do this, we first computed the topic distribution for each of the 15 outlets, detailing the proportion of their news articles which fall into each of our 12 topic categories or the “Other” category, as shown in Fig 3. From this, we computed the expected gender balance for a news outlet based upon its distribution of topic categories by taking the inner product between the outlet's topic distribution and the gender balance of each topic respectively. For each outlet, we compared the expected gender balance given the topics it covers to the actual gender balance we found, as shown in Fig 4.

Our findings show that the gender imbalance in images chosen by Forbes can be explained by its choice of news topics, but the difference in the percentage of females mentioned in the text cannot be attributed to the topics that it covers, with the gender balance we found being 5% more probable to feature a male entity than we would expect based upon topic coverage alone. Conversely, we found that News.com.au shows more gender balance than we would typically expected given its coverage of topics, with the probability that it features a female being 3–4% more than expected given its topic distribution.

## Discussion

Why are women routinely marginalized or “symbolically annihilated” in the news, to utilize a term developed by media sociologist Gaye Tuchman [13]. Over several decades, feminist news research has demonstrated that there is a persistent pattern of underrepresentation of women in relation to men in the world’s news media. Feminist news researchers have argued that the underrepresentation of women in the news undermines important liberal principles, thus undermining democracy itself.

Western, liberal philosophy is based on the assumption that the human mind is separate, in principle, from the human body. Jaggar [12] uses the term ‘normative dualism’ to describe the view that “what is especially valuable about human being is their ‘mental’ capacity for rationality.” For Jaggar, normative dualism is an example of the male bias in western philosophy, where “excessive value is placed on the ‘mind’ at the expense of the body” [12]. This sexual division equates the mind with the masculine and culture and the body with the feminine and nature. In the case of the media representation of men and women in sport, there is a further hierarchical sexual division between the active, performative masculine sporting body and the aesthetic, erotic feminine, passive sporting body. As Rowe [31] suggests, “In potentially opening themselves up to the ‘feminization’ of their body image by being portrayed in a sexualized manner, sportsmen seek to counter such a process by doing something—anything—rather than just receive the gaze.”

Our paper both provides the largest data-driven study to date demonstrating the systematic under-representation of women in the news narrative, and also provides detailed information about the domains in which women are more or less represented. The domains where women are more present include fashion and entertainment, while men are more associated with business and politics. Importantly, women are more often portrayed in images than they are represented in text.

In particular, we have shown how the gender balance in top stories of online news media differs across topics, outlets and modes of content, concluding that females are more likely to be seen than mentioned in a news article. More generally, the news media are still very much male-dominated, with an overall probability of 77.0% that an entity mentioned in the text is male, or 69.6% that a face image is male.

This work therefore provides extensive experimental evidence for the ways in which the (online English language) news consistently and systematically contributes to the reproduction of gender inequalities in quantitative terms and qualitatively.

These findings confirm those of smaller-scale studies performed using traditional analytical techniques, such as that undertaken by Desmond and Danilewicz [32] who examined 580 televised news stories, finding that female reporters were more likely to present human interest and health related stories, and that males were significantly more often used as experts than their female counterparts. Similar results have been reported for printed news media [7,8,9,10,11], with men writing the majority of the news and are most often quoted or mentioned within the news stories. Other large-scale studies using data-driven approaches, such as that undertaken by Wagner et al [33], have shown that gender imbalance is also an issue on the web, finding in a selection of 124,824 articles from Wikipedia that while women were featured equally as often as men, the representation of those women was very different from men, often focussing on romantic and family-related issues.

Automated approaches that combine computer vision and natural language processing technologies with vast data samples make it possible, for the first time, to gather extensive empirical support, on a scale previously inconceivable, for long-standing claims around the marginalisation of women in the news. Facilitating analyses that would otherwise take years to complete, such technologies enable new forms of critical enquiry in this field of research. The importance of such advanced forms of quantitative analysis lies in the ability to clearly document, through enormous, finely detailed datasets, the presence of pernicious, patterned gender inequalities and, indeed, more optimistically, proof of progressive gender representations when and where they occur. As such, they offer hope in efforts to challenge the marginalisation of women’s voices in the news media and in so doing acknowledge the value of their contributions to the future strength of deliberative democracy.

## Supporting Information

S1 Fig Percentage of images in each label category for each topic. Images were extracted for news articles classified into each topic category, with the percentage of the news articles with no image, a missing image, an image that does not contain a face, and face images displayed in the stacked bar chart.
(EPS)

S2 Fig Percentage of images in each label category for each news outlet. Images were extracted from news articles published by each news outlet, with the percentage of news articles with no image, a missing image, an image that does not contain a face, and face images displayed in the stacked bar chart.
(EPS)
